# Supplementary material for: Solubility and Decomposition of Organic Compounds in Subcritical Water
Source: Molecules. 2023 Jan 19;28(3):1000. doi: 10.3390/molecules28031000 (PMC9920662; doi:10.3390/molecules28031000)
Supplement: Supplementary file 1 [file molecules-28-01000-s001.zip › molecules-2140167-supplementary.pdf]

molecules

Supplementary Materials

Solubility and Decomposition of Organic Compounds in Subcritical Water

Erdal Yabalak [1], Sema Akay [2], Berkant Kayan [2], A. Murat Gizir [3,*] and Yu Yang [4,*]

1 Department of Nanotechnology and Advanced Materials, Mersin University, 33343 Mersin, Türkiye
2 Department of Chemistry, Arts & Science Faculty, Aksaray University, 68100 Aksaray, Türkiye
3 Department of Chemistry, Faculty of Science, Mersin University, 33342 Mersin, Türkiye
4 Department of Chemistry, East Carolina University, Greenville, NC 27858, USA
* Correspondence: agizir@mersin.edu.tr (A.M.G.); yangy@ecu.edu (Y.Y.)

Table S1. Carbohydrates Solubilities in Subcritical Water.

Compound	Temperature range (K)	Pressure (bar)	Medium/Solvent	Solubility range (x_2)[a]	Empirical equations	Reference
Glucose	293–453	N/A	Subcritical water	$4.45\times10^{-2} – 2.32\times10^{-1}$	The modified Apelblat equation or the A-UNIFAC model	[52]
Maltose				$2.44\times10^{-2} – 1.47\times10^{-1}$		
Xylose				$5.08\times10^{-2} – 2.07\times10^{-1}$		

[a] Mole fraction solubility of compounds.; N/A: Not Available

Table S2. Preservative Ingredient Solubilities in Subcritical Water.

Compound	Temperature range (K)	Pressure (bar)	Medium/Solvent	Solubility range (x_2)[a]	Empirical equations	Reference
Methyl paraben	298–473	5–35	Subcritical water	$2.50\times10^{-4} – 1.50\times10^{-3}$	$\ln x_2 = \left(\frac{T_o}{T}\right)\ln x_2(T_o) + 0.5(C-1)\left(\frac{T_o}{T}-1\right)$	[54]
Ethyl paraben				$0.74\times10^{-4} – 0.91\times10^{-3}$		
Butyl paraben				$0.18\times10^{-4} – 0.41\times10^{-3}$		

[a] Mole fraction solubility of compounds.
